# Evaluating the synergistic effects of cisplatin and tamoxifen in canine osteosarcoma cells

**DOI:** 10.3389/fvets.2026.1802456

**Published:** 2026-06-05

**Authors:** Marshall Mays, Kayden Tanner, Tomas Lugo, Thu Annelise Nguyen

**Affiliations:** School of Veterinary Medicine, Texas Tech University, Amarillo, TX, United States

**Keywords:** cancer, cancer treatment, canine, cisplatin, combinational therapeutic strategy, osteosarcoma, synergy, tamoxifen

## Abstract

**Introduction:**

Canine osteosarcoma is an aggressive primary bone tumor with a high metastatic rate and poor long-term prognosis. Therapeutic resistance remains a major cause of treatment failure. In human osteosarcoma, sex hormones influence tumor survival and proliferation, and emerging evidence suggests a similar role in canine osteosarcoma. Tamoxifen, a selective estrogen receptor modulator, inhibits estrogen receptor signaling and also exhibits estrogen-independent effects, including suppression of multidrug resistance pathways. Cisplatin is commonly used in canine osteosarcoma treatment; however, resistance limits its effectiveness. Therefore, we hypothesized that tamoxifen could enhance cisplatin efficacy through estrogen-independent mechanisms.

**Methods:**

Canine osteosarcoma cells were treated with a matrix of tamoxifen and cisplatin concentrations. Cell viability was assessed at 12, 24, and 48 hours using metabolic assays. Cleaved caspase activation and apoptosis-inducing factor expression were evaluated as markers of cell death, while Cyclin D1 and Ki-67 were assessed as markers of cell proliferation. Drug interaction was analyzed using the Zero Interaction Potency model.

**Results:**

Synergy analysis at 24 hours demonstrated strong synergistic activity between tamoxifen and cisplatin, with a ZIP score of 16.82. Combination treatment reduced metabolic activity and activated apoptotic markers at lower drug concentrations compared with either treatment alone. Although estrogen receptor expression was confirmed, the enhanced cytotoxic effect of tamoxifen appeared to be largely mediated through estrogen-independent mechanisms.

**Discussion:**

These findings suggest that tamoxifen enhances cisplatin-based combination therapy by promoting cell death in canine osteosarcoma at reduced drug concentrations. Combination therapies targeting estrogen-independent pathways may represent a promising strategy for improving treatment response in metastatic canine osteosarcoma and warrant further investigation.

## 1 Introduction

Osteosarcoma (OSA) is currently the most common primary bone tumor in dogs, accounting for roughly 85% of all of skeletal tumors (1). Current literature estimates that approximately 8,000 to 10,000 dogs are diagnosed with OSA each year (2, 3). OSA is a malignant bone cancer arising from abnormal production of osteoblasts and osteoclasts most commonly affecting the long bones of the forelimbs or hindlimbs (4). Common clinical signs include pain, swelling, and lameness, resulting from tumor induced destruction of normal bone and replacement with neoplastic tissue commonly leading to pathological fractures (1, 5, 6). Of greater concern, OSA is highly aggressive, with micrometastases reported to be present at the time of diagnosis in 90% of cases, most frequently involving the lungs (1, 4).

Currently, there is no curative treatment for osteosarcoma and management is palliative in nature with the goal of improving quality of life (6). The current standard of care typically includes pain management, surgical intervention, and adjuvant chemotherapy (4, 6, 7). Amputation or limb sparing surgery without adjuvant chemotherapy is associated with median survival times of only 3–4 months and carries a substantial risk of tumor recurrence, often leading to euthanasia or death (1, 4). Surgical removal is typically followed by adjuvant chemotherapy, which provides only limited benefit in metastatic disease (8–11). Even with chemotherapy, survival outcomes for dogs with metastatic osteosarcoma remain poor, with approximately 60% surviving 1 year and only 10% surviving 3 years (8–10, 12).

Common chemotherapeutic agents used for canine osteosarcoma include cisplatin and carboplatin (5, 13). Recent literature recommends carboplatin over cisplatin clinically due to the nephrotoxicity, nausea, and gastrointestinal risks associated with cisplatin (1, 14). However, cisplatin remains a potent antitumor agent, and there is no clear evidence that either drug is more effective (1). In practice, achieving therapeutic efficacy with cisplatin may require doses that exceed patient tolerability. This is partially driven by the tumor's intrinsic and acquired resistance often requiring higher chemotherapy doses that worsen side effects (13).

While chemoresistance is rarely driven by one factor, recent studies have characterized osteosarcoma tumors, identifying proteins and genes that promote tumor survival and resistance to apoptosis. One example is the membrane transporter ATP-binding cassette subfamily B member 1 (ABCB1), also known as multidrug resistance 1 (MDR1). MDR1 encodes P-glycoprotein which acts as an efflux pump and is frequently overexpressed in osteosarcoma, contributing to chemoresistance (15–18). Key signaling pathways include Insulin-like Growth Factor (IGF-1/IGF-1R), Transforming Growth Factor-β (TGF-β), Protein Kinase C (PKC), IGF-1/IGF-1R, and TGF-β (15, 19, 20). Elevated activity of these mechanisms and pathways in both human and canine osteosarcoma highlights potential therapeutic targets. Although these specific pathways and proteins were not directly evaluated in this cell line, prior literature provides a rationale for therapeutic strategies aimed at mitigating cisplatin's dose limiting toxicities.

Recent studies report promising results and increasing interest in interventions that enhance chemotherapy efficacy while potentially targeting key tumor survival pathways (21–24). One example is tamoxifen, a selective estrogen receptor modulator that has been shown to enhance the efficacy of conventional chemotherapeutics in human osteosarcoma and breast cancer cell line models (21, 25, 26). Tamoxifen can exhibit both estrogen dependent and independent anticancer effects. Tamoxifen has also shown to inhibit important resistance mechanisms and survival pathways, suggesting potential utility in combination therapy for dogs, though canine clinical studies remain limited (16, 17, 21, 27–29).

In this study, we investigated whether combining tamoxifen and cisplatin at reduced concentrations could achieve anticancer effects comparable to standard single-drug therapy. We hypothesized that tamoxifen would synergistically enhance cisplatin's activity, providing a rationale for combination therapy in veterinary oncology. To test this, we used a metastatic canine osteosarcoma model derived from an 11-year-old poodle. Cells were treated with cisplatin, tamoxifen, or combinations of both across a range of concentrations. Viability assays, western blotting, and immunofluorescence were performed to assess cellular responses and further understand the potential drug mechanisms.

To evaluate true pharmacologic synergy, we systematically evaluated cisplatin and tamoxifen combinations across a range of concentrations and quantified their interaction using the zero-interaction potency (ZIP) model. The ZIP model first defines an expected response assuming no interaction between the two drugs. The observed combination effects are then compared with this reference and summarized as ZIP synergy scores, where positive values indicate synergy, values near zero reflect additivity, and negative values indicate antagonism (30, 31). By applying this model, we systematically evaluated whether the cisplatin-tamoxifen combination exhibits true pharmacologic synergy or merely additive effects.

## 2 Methods

### 2.1 Cells and cell culture

D-17 CCL-183 canine osteosarcoma cell line was obtained from American Type Culture Collection (ATCC, Manassas, VA, USA). Cells were cultured in Roswell Park Memorial Institute Medium 1640 (RPMI 1640 Medium; Gibco; Thermo Fisher Scientific, Inc., Waltham, MA, USA) containing 10% fetal bovine serum (FBS; Invitrogen; Thermo Fisher Scientific, Inc., Waltham, MA, USA) at 37 °C in a humidified incubator with 5% CO_2_. To minimize bacterial and fungal contamination, 1% antibiotic-antimycotic solution (100 × Antibiotic-Antimycotic; Gibco, Thermo Fisher Scientific, Waltham, MA, USA) was added to the culture medium. All experiments were conducted using cells between passages 30 and 35. Cells were not used beyond passage 35, and experiments were restarted from cryopreserved stocks at passage 30 to maintain consistency.

### 2.2 Drugs and drug treatment

D-17 osteosarcoma cells were exposed to tamoxifen and cisplatin either as single agents or in combination. Tamoxifen (Sigma-Aldrich, Cat. No 579000, ≥99% purity) was dissolved in dimethyl sulfoxide (DMSO; MilliporeSigma, Sigma-Aldrich; Cat. No. D8418-50 ML) to prepare a concentrated stock solution (10 mM), aliquoted, and stored at −20 °C, protected from light. Cisplatin (Sigma-Aldrich, Cat. No. 232120, ≥98% purity) was dissolved in sterile 0.9% NaCl according to the manufacturer's instructions to obtain a stock solution (10 mM), aliquoted, and stored at −20 °C. Immediately before each experiment, working concentrations were prepared by diluting stock solutions into complete RPMI-1640 medium; the final DMSO concentration in culture never exceeded 0.1%.

For viability, western blot analysis and immunofluorescence assays (IFA), cells were seeded at appropriate densities of 10,000 cells/well in 96-well plates, 50,000 cells/well in 12-well plates, and 2 million cells per T-75 cm^2^ flasks, respectively, for protein extraction and allowed to adhere for 24 h. Cells were then treated for the indicated durations up to 48 h with a range of tamoxifen and cisplatin concentrations, either alone or in ratio combinations. Vehicle treated cells with RPMI media containing 0.1% DMSO served as controls. After the treatment period, cells were processed for RealTime-Glo viability measurements, protein extraction for western blotting, or fixation and staining for IFA.

Additionally, different treatment durations were selected for downstream assays to capture distinct biological endpoints. Viability assays were performed continuously up to 48 h to assess cumulative cytotoxic effects, whereas western blot and immunofluorescence analyses were conducted at 24 h to evaluate early apoptotic and cell cycle signaling events prior to extensive cell death or detachment observed at later time points.

### 2.3 Quantitative analysis of cell metabolic viability

Cell viability was assessed using the RealTime-Glo™ MT Cell Viability Assay (Promega, Madison, WI, USA; Cat. No. G9711) according to the manufacturer's instructions with minor modifications. D-17 cells were plated in white-walled, clear-bottom 96-well plates at 10,000 cells/well in 200 μL complete RPMI-1640 media with 10% FBS and allowed to attach for 24 h. Cells were then exposed to tamoxifen or cisplatin at the indicated concentrations. Cisplatin was evaluated across a range of concentrations, including 100, 250, 500, 750, 1,000, 1,250, and 1,500 μM. Tamoxifen was tested over a concentration range of 10, 25, 50, 65, 75, 85, and 100 μM. Vehicle-only wells containing 0.1% DMSO served as negative controls, and cell-free wells containing medium supplemented with RealTime-Glo reagent were included for background subtraction.

Immediately before treatment, the RealTime-Glo substrate enzyme mixture was prepared and added to each well at the recommended dilution of 2 × per well. Luminescence, reflecting metabolically active cells, was measured at baseline and at 1-h intervals up to 48 h using a BioTek Cytation 5 Cell Imaging Multimode Reader with environmental control maintained at 37 °C and 5% CO_2_. Background-corrected luminescence values were normalized to vehicle controls to determine relative viability at each concentration and time point, and this data was subsequently used to calculate LD50 values for single-agent treatments. Measurements were collected with six technical replicates per condition across three independent biological replicates.

Prior to combination treatment experiments, the half-maximal lethal dose (LD50) for tamoxifen and cisplatin was first determined individually using Hill equation of four-parameter logistic or commonly known as the sigmoid Emax dose-response curve (LL.4) (32). In the four-parameter log-logistic model, the response () is modeled as a function of time (*X*). The parameters *a* and *b* represent the lower and upper values of the response, respectively, *d* defines hill's slope of the curve, and *c* corresponds to the effective dose producing a half-lethal dose (LD50). This equation was used in R studio to extrapolate the LD_50_ values (33, 34).


$$\begin{array}{l} \gamma =a+\frac{b-a}{1+{\left(\frac{c}{X}\right)}^{d}} \end{array}$$

### 2.4 Combination ratios

Based on the resulting LD50 values, a full factorial 5 × 5 drug combination matrix was designed to systematically evaluate drug-drug interactions. Tamoxifen was tested at 0, 26, 52, 78, and 156 μM, while Cisplatin was tested at 0, 75.5, 151, 226.5, and 453 μM. All pairwise combinations of the selected concentrations were tested, yielding 25 distinct treatment conditions. Each condition was run in technical triplicates, with remaining wells allocated to vehicle and untreated controls. The full experiment was repeated across two independent biological replicates. This factorial layout supported quantitative synergy assessment over a broad dose landscape using ZIP modeling.

Immunofluorescence analysis (IFA) and western blot analysis were performed using the 24 h LD50 combination extrapolated from RealTime-Glo viability assay values and defined pairwise treatment conditions. For IFA, cells were treated with the LD50 combination and four additional pairwise combinations, LD50 (74 μM tamoxifen and 240 μM cisplatin), combination 1 (75 μM tamoxifen and 75 μM cisplatin), and combination 2 (26 μM tamoxifen and 151 μM cisplatin).

Western blot analysis included the LD50 condition and six additional pairwise combinations: combination 1 (75 μM tamoxifen and 75 μM cisplatin), combination 2 (52 μM tamoxifen and 151 μM cisplatin), combination 3 (52 μM tamoxifen and 226 μM cisplatin), combination 4 (52 μM tamoxifen and 453 μM cisplatin), combination 5 (26 μM tamoxifen and 75 μM cisplatin), and combination 6 (26 μM tamoxifen and 151 μM cisplatin).

### 2.5 ZIP model

Tamoxifen and cisplatin interactions were quantified using the Zero Interaction Potency (ZIP) model implemented in SynergyFinder software: https://doi.org/10.1093/nar/gkac382 (35). Normalized RealTime-Glo™ viability values were divided based on the vehicle control was averaged across technical replicates for each condition along with time points and imported into SynergyFinder as a 5 × 5 dose–response table. The ZIP reference model compares the observed combination effect to the expected response under the assumption that combining drugs does not alter their individual potencies. For each time point, SynergyFinder generated a global ZIP synergy score for the full matrix and local ZIP scores visualized as two-dimensional synergy landscapes and dose–response curves.

### 2.6 Immunofluorescence assay

Immunofluorescence assays were performed to assess the qualitative expression levels and protein staining patterns of caspase-3, Ki-67, ERα66, and apoptosis-inducing factor (AIF). Cells were seeded onto coated coverslips in 24-well plates (Nunc™ Thermanox™; Thermo Fisher Scientific) and cultured in RPMI at 37 °C, 5% CO_2_ to ~70% confluency. Cells were washed 3 × with Phosphate-buffered saline (PBS), fixed with 4% paraformaldehyde (20 min, room temperature), washed 3 × with PBS, permeabilized with 0.25% Triton X-100 (20 min, room temperature), washed 3 × with PBS, and blocked with Intercept Blocking Buffer (2 h, room temperature). Coverslips were incubated overnight at 4 °C with primary antibodies (Cleaved caspase-3/caspase-3; Invitrogen, CAT# 43-7800, Lot# 3214139; Ki-67; Invitrogen, Cat# MA5-14520, Lot# 79641269; Estrogen Receptor alpha; Cell Signaling Technology, Cat# 8644S, Lot# 10; Apoptosis-Inducing Factor; Cell Signaling Technology, Cat# 4642S, Lot# 3; Vimentin; Invitrogen, Cat# MA5-11883, Lot# 3138686 and Cat# PA5-143592, Lot# 7969773) 1:500 in blocking buffer), washed 3 × with PBS.

Following washing, coverslips were incubated with Alexa Fluor conjugated secondary antibodies (goat anti-rabbit IgG [H+L], Invitrogen, Cat# A21202, Lot# 2147618, Alexa Fluor 488; goat anti-rabbit IgG [H+L], Invitrogen, Cat# A11037, Lot# 2160403, Alexa Fluor 594; donkey anti-mouse IgG [H+L], Invitrogen, Cat# A21209, Lot# 2273768, Alexa Fluor 594; goat anti-mouse IgG [H+L], Invitrogen, Cat# A11029, Lot# 840881, Alexa Fluor 488) in 1:1,000 with blocking buffer (2 h, Room Temperature). Nuclei were counterstained with DAPI (1:1000, 10 min), followed by a final wash, and coverslips were mounted using ProLong™ Gold Antifade Mountant (Invitrogen; Thermo Fisher Scientific, Inc., Waltham, MA, USA). All images were acquired on a Revolution automated fluorescence microscope using a 40 × objective and the DAPI, FITC, and TRITC channels. For each channel, exposure settings were held constant across all samples and were established using negative controls to define background. All immunofluorescence assay experiments were conducted using three independent biological replicates, with each replicate representing a complete, independently performed experiment, including cell treatment, fixation, staining, antibody incubation, and imaging.

Ki-67 was assessed by immunofluorescence because it is a well-established nuclear marker of cellular proliferation, expressed during almost all active phases of the cell cycle, making it useful for qualitative visualization of proliferative activity in cells. However, Ki-67 was not used for western blot quantification due to technical limitations associated with its large molecular weight (approximately 345–395 kDa, species-dependent), which can make consistent detection by western blot challenging. Cyclin D1 was selected for western blot analysis as a complementary marker of cell cycle progression, as it is a lower-molecular-weight protein (~36 kDa) that is more reliably detected and quantified.

Vimentin was included in early immunofluorescence experiments as an internal control to confirm appropriate antibody binding and staining conditions. Once antibody performance was confirmed, vimentin was no longer routinely included in all subsequent assays. Immunofluorescence was used solely as a qualitative visualization tool to assess expression patterns and cellular localization, whereas all quantitative protein expression analyses were performed using western blotting.

### 2.7 Western blot

Western blotting was performed to assess quantitative expression levels of caspase-3, cyclin D1, ERα66, Apoptosis-Inducing Factor (AIF), and β-actin used as the control marker. Cells were washed with PBS and lysed in RIPA buffer supplemented with protease and phosphatase inhibitors on ice. Lysates were sonicated and separated by centrifugation at 4 °C, and protein concentration was determined using a bicinchoninic acid (BCA) assay (Pierce™ BCA Protein Assay Kit, Thermo Fisher Scientific, Waltham, MA, USA). Equal amounts of total protein were mixed with 4 × Laemmli sample buffer containing reducing agent, denatured, and separated by SDS–PAGE, then transferred to a PVDF membrane. Membranes were blocked in 5% non-fat milk (or 5% BSA, as appropriate) for 30 min in Tris-Buffered Saline with 0.1% Tween-20 (TBST) and incubated overnight at 4 °C with primary antibodies (Cleaved caspase-3/caspase-3; Invitrogen, CAT# 43-7800, Lot# 3214139; cyclin D1; Cell Signaling Technology, Cat# 2978, Lot# 8; estrogen receptor alpha [ERα66]; Cell Signaling Technology, Cat# 8644S, Lot#10; Apoptosis-Inducing Factor; Cell Signaling Technology, Cat# 4642S, Lot# 3; β-actin; Cell Signaling Technology, Cat# 4970, Lot# 49705) with TBST at 1:500 ratio.

**Figure 1:**
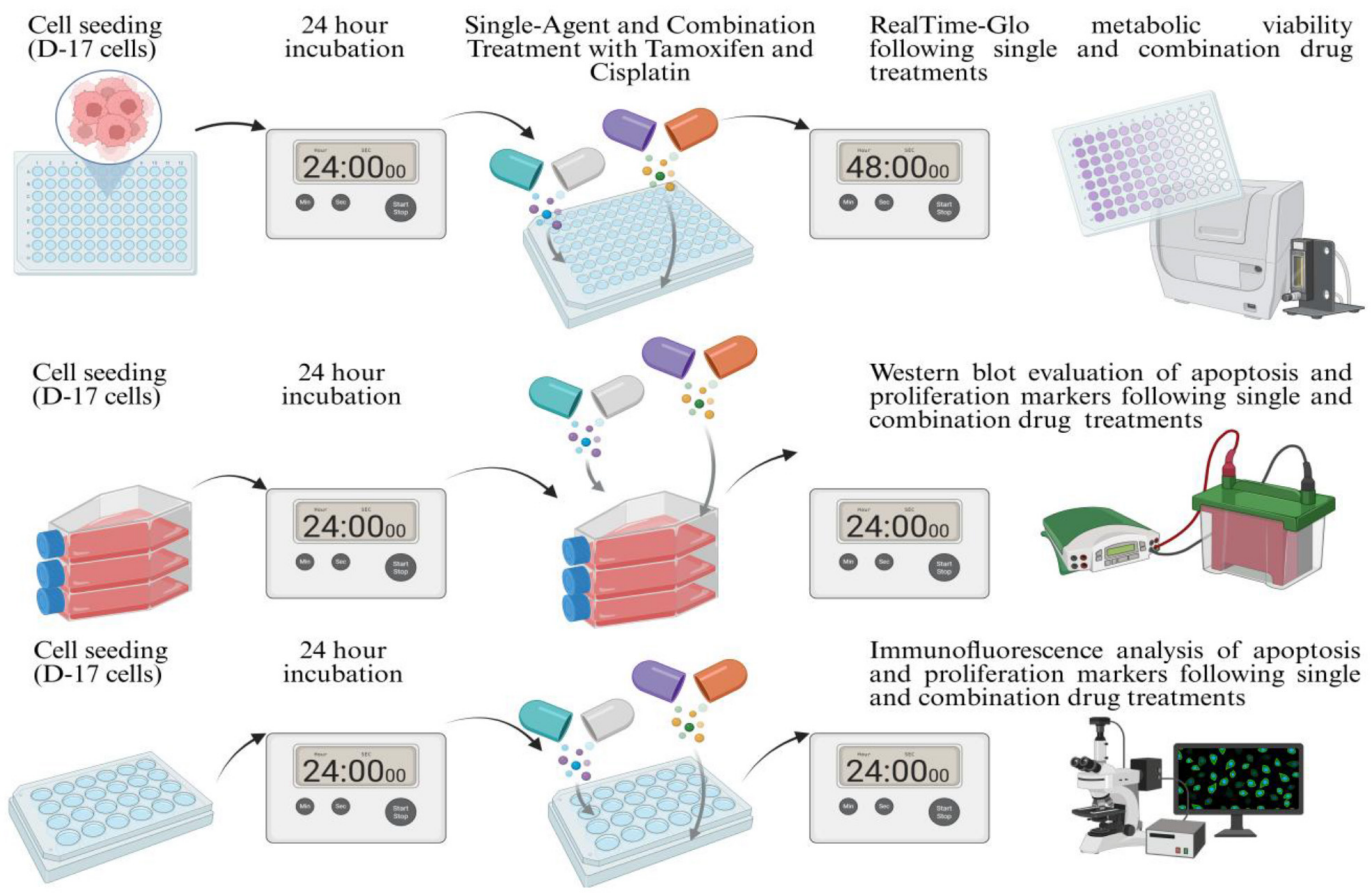
Schematic workflow for D-17 cell experiments. D-17 cells were seeded and incubated for 24 h, then treated with tamoxifen, cisplatin, or their combination. Cell viability was monitored continuously for up to 48 h using the RealTime-Glo™ viability assay, while western blot and immunofluorescence analyses were performed at 24 h to assess apoptotic and proliferative markers. Created in BioRender. Lugo, T. (2026) https://BioRender.com/3n7qf9z.

After washing in TBST, membranes were incubated with HRP-conjugated anti-rabbit IgG secondary antibody (Cell Signaling Technology, Cat# 7074P2, Lot#34) and developed using enhanced chemiluminescence (ECL). Band intensities were quantified and normalized to β-actin as a loading control using U-SCAN-IT gel version 7.1v. For caspase-3 and AIF, both full-length and cleaved forms were assessed when applicable. All western blot experiments were conducted using three independent biological replicates, with each replicate representing a separate experiment performed in its entirety, including cell treatment, protein extraction, electrophoresis, membrane transfer, antibody incubation and image analysis. The methodological workflow is illustrated in Figure 1.

### 2.8 Statistical analysis

Western blot densitometry data were analyzed using a repeated-measures statistical framework to account for paired measurements obtained from the same blot across treatment conditions. Normalized protein expression values were summarized as average ± standard error of the mean across independent blots. To assess treatment-dependent effects, a repeated measures analysis of variance (ANOVA) was performed that included treatment concentration as a fixed effect and blot identifier as a factor to control for within-blot variability. When a significant overall treatment effect was detected, *post-hoc* multiple comparisons were conducted using Dunnett's test to compare each treatment condition directly against the corresponding untreated control (0 μM). Statistical significance was defined as an adjusted *p*-value < 0.05 (^*^) and *p* < 0.01 (^**^). All statistical analyses and graphical representations were performed in R (version 4.3.3).

## 3 Results

### 3.1 Metabolic activity dose–response of cisplatin and tamoxifen

**Figure 2:**
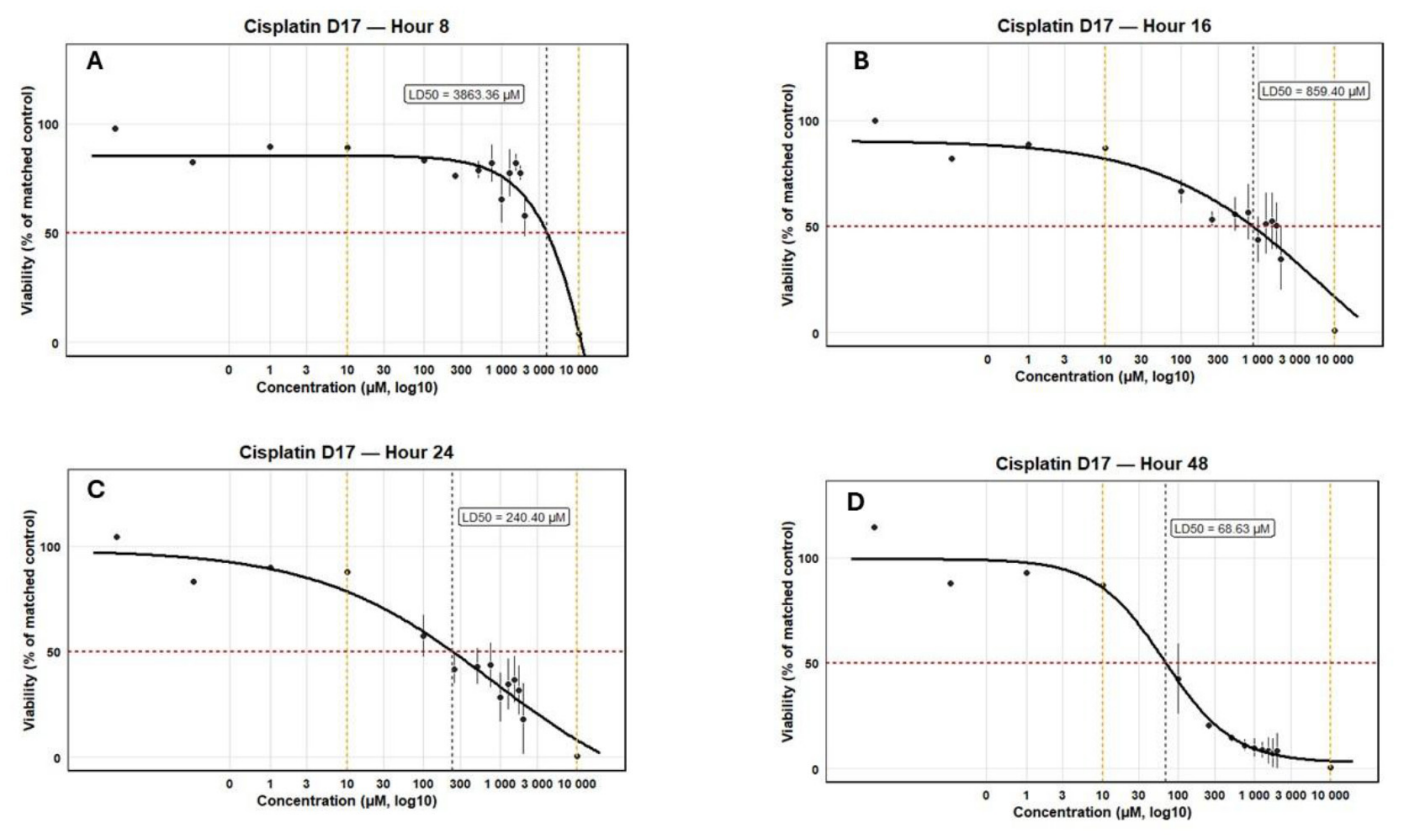
Cisplatin dose–response in D17 cells over time (8, 16, 24, 48 h) which reflects **(A–D)**, respectfully. Viability (%) is plotted vs concentration (μM, log_10_). Points are biological means (6 technical replicates per biological) with error bars (SEM) Black curves show LL.4 fits; LD_50_ is the concentration at 50% viability (horizontal red dashed line) and is indicated by the vertical dashed line/label. Yellow dashed lines (10 and 10,000 μM) mark the initial *n* = 1 screening bounds used to bracket the LD_50_ window, followed by increased replication (biological *n* = 3) to refine the estimate.

Metabolic activity following cisplatin exposure was evaluated in D-17 cells using RealTime-Glo viability assay at 8, 16, 24, and 48 h (Figure 2). Cisplatin was administered as individual doses ranging from 10 nM to 10,000 μM, with additional triplicate measurements performed between 100 and 1,500 μM to improve LD_50_ range. The estimated LD50 values were approximately 3,863.36 μM at 8 h, 859.40 μM at 16 h, 240.40 μM at 24 h, and 68.63 μM at 48 h. A decrease in viability was seen with each increasing dosage.

**Figure 3:**
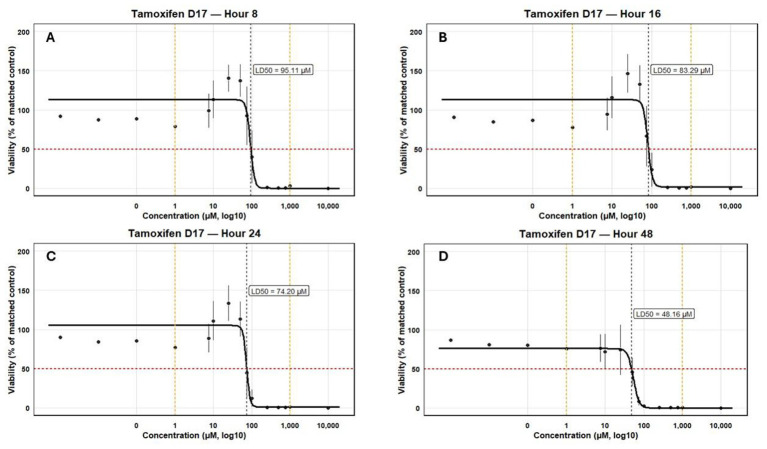
Tamoxifen dose response in D17 cells over time (8, 16, 24, 48 h) which reflects **(A–D)**, respectfully). Viability (%) is plotted vs. concentration (μM, log_10_). Points are biological means (6 technical replicates per biological) with error bars (SEM). Black curves show LL.4 fits; LD_50_ is the concentration at 50% viability (horizontal red dashed line) and is indicated by the vertical dashed line/label. Yellow dashed lines (1 and 1,000 μM) mark the initial *n* = 1 screening bounds used to bracket the LD_50_ window, followed by increased replication (biological *n* = 3) to refine the estimate.

Tamoxifen-induced metabolic activity in D-17 cells was assessed using RealTime-Glo viability assay at 8, 16, 24, and 48 h (Figure 3). Cells were treated with tamoxifen at concentrations ranging from 10 nM to 10,000 μM, with additional triplicate measurements within 10 μM to 100 μM to better define the LD50. The estimated LD50 values were 95.11 μM at 8 h, 83.29 μM at 16 h, 74.20 μM at 24 h, and 48.16 μM at 48 h. Across all time points, increasing tamoxifen concentrations corresponded to progressively lower cell viability.

### 3.2 ZIP model analysis of Cisplatin–Tamoxifen combination therapy

**Figure 4:**
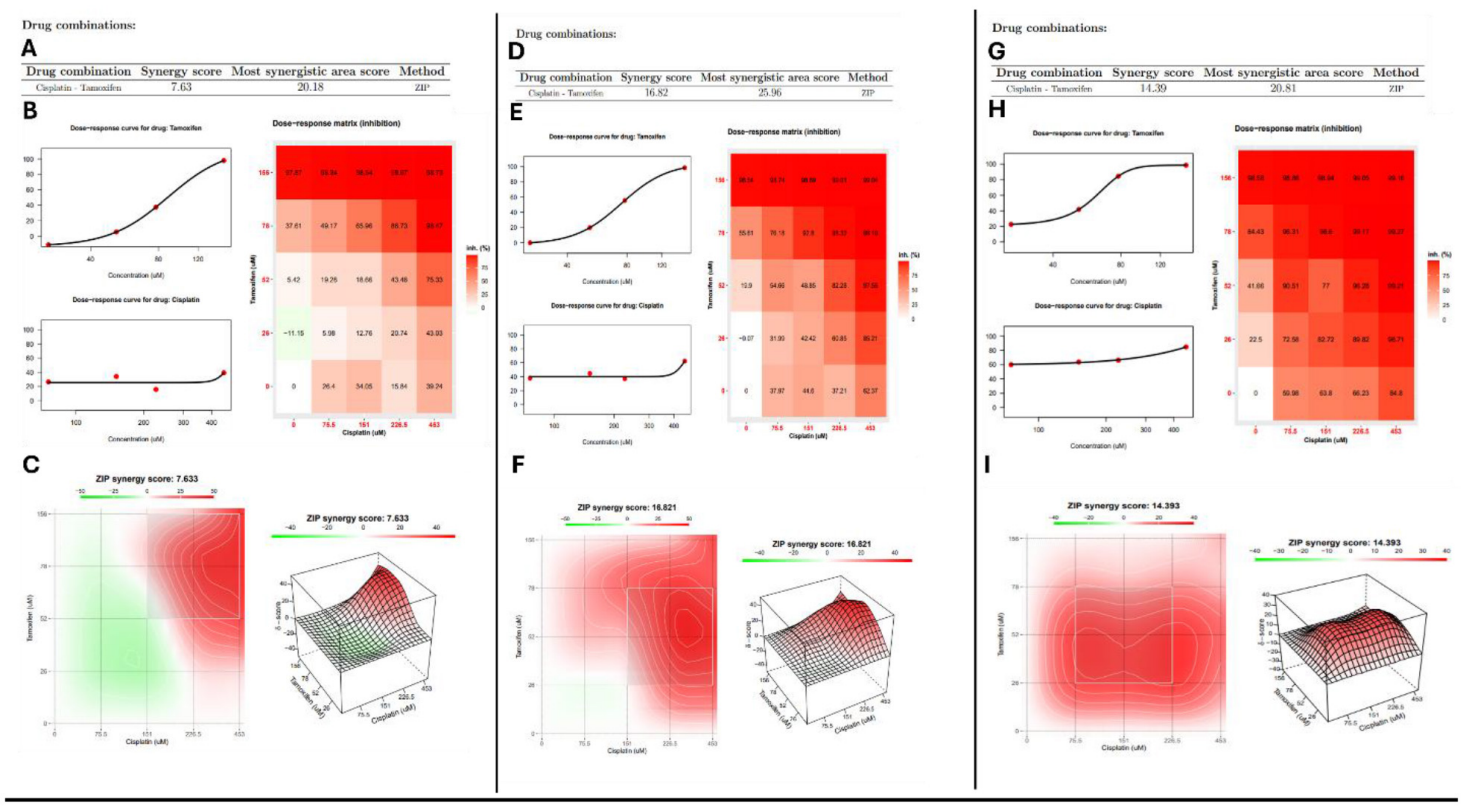
ZIP synergy analysis for the cisplatin and tamoxifen combination at 12 h **(A–C)**, 24 h **(D–F)**, and 48 h **(G–I)**. For each time point, summary tables **(top)** report the overall ZIP synergy score and most synergistic region, single-drug dose–response curves with the inhibition matrix are shown **(middle)**, and ZIP synergy heatmaps with 3D synergy surfaces are shown **(bottom)**.

Combination therapy with cisplatin and tamoxifen demonstrated a mean ZIP synergy score of 7.633 at 12 h, with a maximum synergistic area score of 20.18 (Figure 4). At 24 h, the mean synergy score increased to 16.82, with the most synergistic area reaching 25.96. At 48 h, the combination maintained a mean synergy score of 14.39, with a maximum synergistic area score of 20.81. At 12 h, regions of positive synergy were more limited within the dose–response matrix compared with 24 and 48 h. By 24 h, the area of positive ZIP scores expanded across a broader range of cisplatin and tamoxifen concentrations, extending toward lower doses of both cisplatin and tamoxifen. At 48 h, positive synergy remained evident across much of the evaluated concentration space, with a more uniform distribution of elevated ZIP scores compared with earlier time points.

### 3.3 Immunofluorescence evaluation of biomarkers following cisplatin and tamoxifen treatment

**Figure 5:**
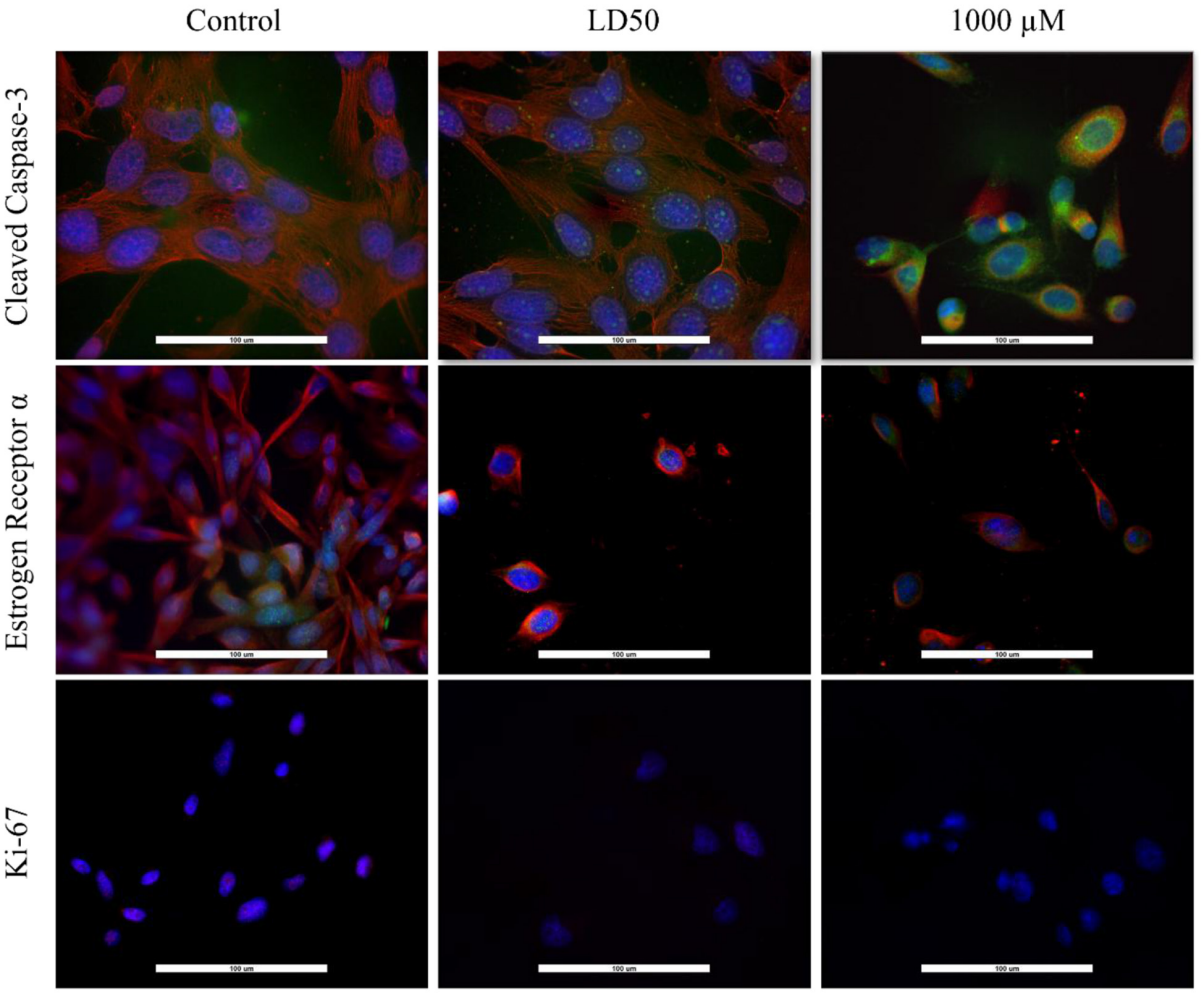
Immunofluorescence analysis of D-17 cells treated with cisplatin. Untreated cells (left column) are shown as controls. Cells were then treated with LD50 cisplatin (middle column) or 1,000 μM cisplatin (right column) for 24 h. Cells were stained for cleaved caspase-3 (cCasp3; top row, green) and estrogen receptor α (ERα; middle row, green) with vimentin (red), and Ki-67 (Ki-67; bottom row, red) with vimentin (green). Nuclei were counterstained with DAPI (miBlue, blue) in all panels. Images were acquired using a 40 × objective. Colors: blue, DAPI (miBlue); green, Green 488; red, Alexa Fluor 594. Scale bar = 100 μm.

Immunofluorescence assays performed on D-17 cells treated with cisplatin showed no observable cleaved caspase-3 signal in control cells, whereas cells treated with 240 μM (LD_50_) and 1,000 μM cisplatin exhibited a visual increase in cleaved caspase-3 activation, with staining observed in both the perinuclear and nuclear areas of the cell. Cisplatin treated cells also displayed cell shrinkage. ERα remained predominantly nuclear and perinuclear after treatment, with little noticeable change in expression compared with controls. Ki-67 showed a general overall decrease in staining intensity in cisplatin treated cells. These observations are shown in Figure 5.

**Figure 6:**
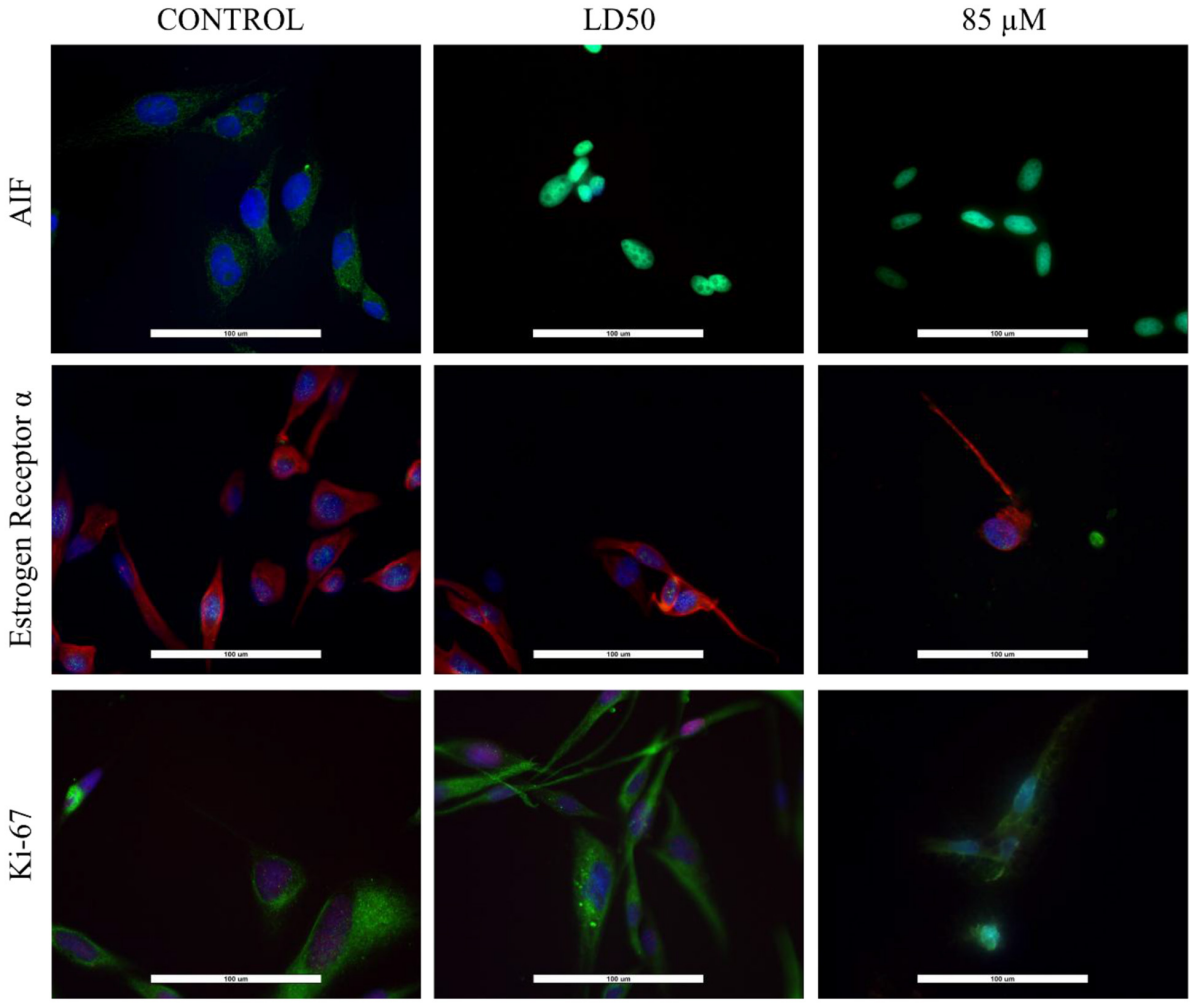
Immunofluorescence analysis of D-17 cells treated with tamoxifen. Untreated cells (left column) are shown as controls. Cells were then treated with LD50 tamoxifen (middle column) or 85 μM tamoxifen (right column) for 24 h. Cells were stained for apoptosis-inducing factor (AIF; top row, green), estrogen receptor α (ERα; third row, green) with vimentin (red), and Ki-67 (Ki-67; bottom row, red) with vimentin (green). Nuclei were counterstained with DAPI (miBlue, blue) in all panels. Images were acquired using a 40 × objective. Colors: blue, DAPI (miBlue); green, Green 488; red, Alexa Fluor 594. Scale bar = 100 μm.

In untreated D-17 cells, AIF was largely confined to the cytoplasm. Treatment with tamoxifen at 75 μM and 85 μM caused AIF to translocate to the nucleus. The nuclear and perinuclear localization of ERα remained largely unchanged following tamoxifen exposure. In contrast, Ki-67 staining was visually reduced in 75 μM (LD_50_) tamoxifen-treated cells with no expression seen in 85 μM. These observations are presented in Figure 6.

**Figure 7:**
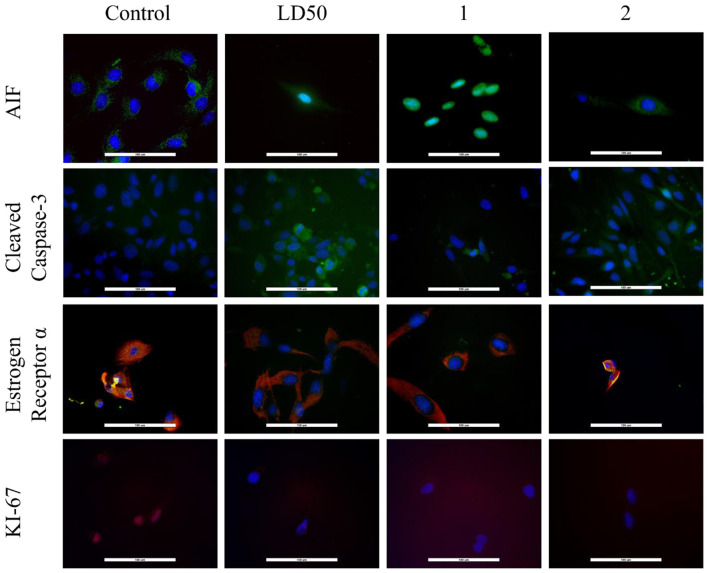
Immunofluorescence analysis of D-17 cells treated with cisplatin and tamoxifen in combination. Untreated cells (left column) are shown as controls. Cells were then treated for 24 h with a combination of cisplatin and tamoxifen at their individually extrapolated LD50 values determined by the RealTime-Glo™ viability assay (second column), combination dose 1 (75 μM cisplatin + 75 μM tamoxifen; third column), or combination dose 2 (151 μM cisplatin + 26 μM tamoxifen; right column). Cells were stained for apoptosis-inducing factor (AIF; top row, green), cleaved caspase-3 (cCasp3; second row, green), estrogen receptor α (ERα; third row, green) with vimentin (red), and Ki-67 (fourth row, red). Nuclei were counterstained with DAPI (blue) in all panels. Images were acquired using a 40 × objective. Colors: blue, DAPI; green, Alexa Fluor 488; red, Alexa Fluor 594. Scale bar = 100 μm.

Combined treatment with cisplatin and tamoxifen produced distinct visual changes in apoptotic and proliferative markers in D-17 cells. In control cells, AIF was predominantly localized to the mitochondria, whereas combination treated cells exhibited translocation of AIF toward the nucleus. This pattern was especially observed in cells treated with the higher tamoxifen doses in combination 1. Cleaved caspase-3 staining was not observed in control cells and visually increased the most following treatment with the LD50 combination. A moderate level of cleaved caspase-3 activation was observed in combination 2, with staining localized primarily to the cytoplasm and evidence of early nuclear translocation. ERα remained largely nuclear and perinuclear across all treatment groups. A general observable decrease in vimentin staining occurred in the treated cells. Ki-67 staining demonstrated an overall visual reduction in proliferative signal in all combination-treated groups compared with controls, with minimal differences between individual treatment conditions. These findings are shown in Figure 7.

### 3.4 Differential quantitation of biomarker expression following cisplatin and tamoxifen treatment

**Figure 8:**
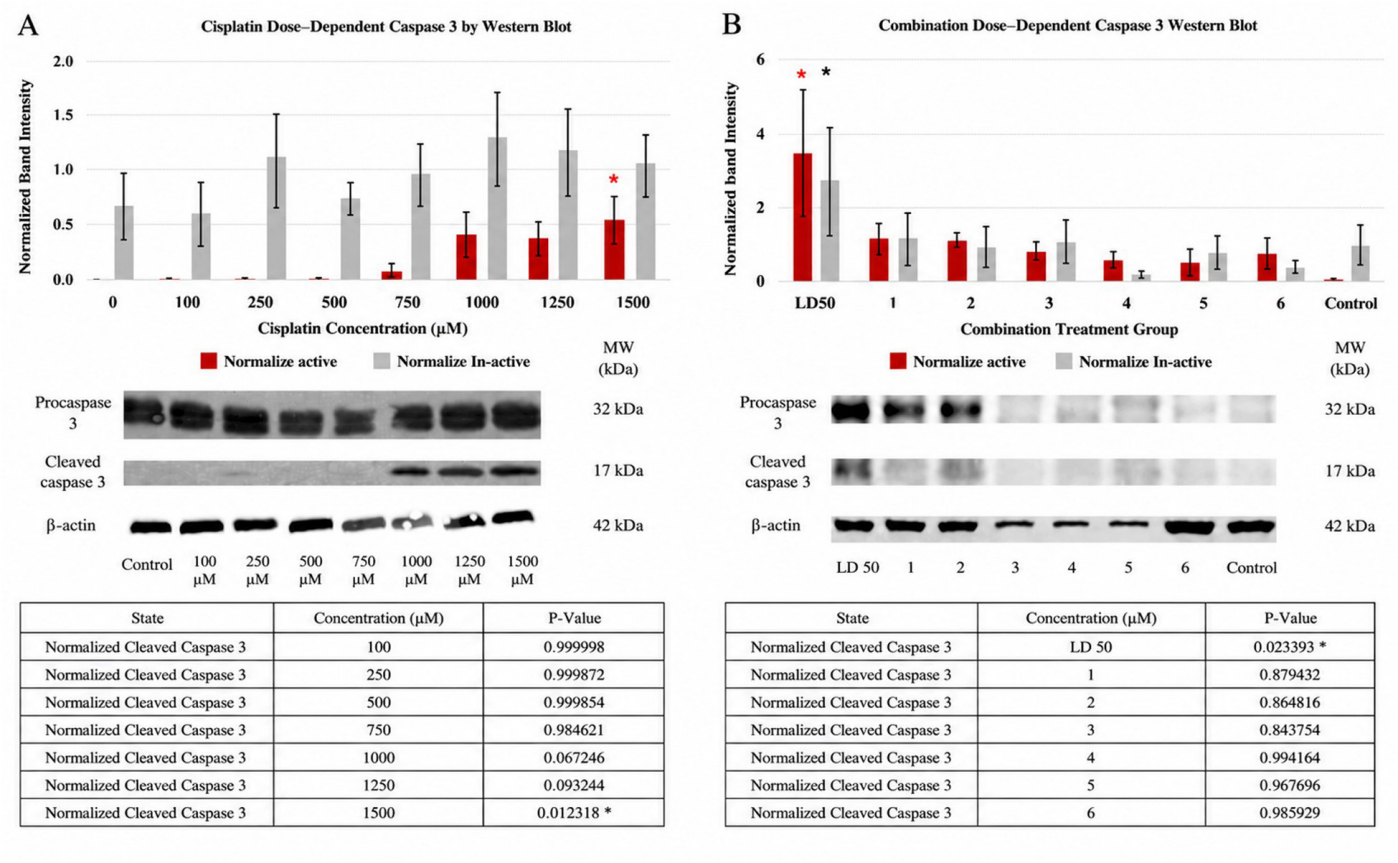
Effects of cisplatin and in combination with tamoxifen on caspase expression in D-17 cells. Cells were treated with cisplatin alone or with cisplatin and tamoxifen for 24 h. Untreated cells served as controls. Western blot analysis was performed to assess pro-caspase-3 and cleaved caspase-3 expression, with β-actin used as a loading control. **(A)** Representative western blots and densitometric analysis of pro-caspase-3 and cleaved caspase-3 following cisplatin treatment alone. **(B)** Representative western blots and densitometric analysis following combination treatment with cisplatin and tamoxifen. Band intensities were quantified using U-Scan-It Gel 7.1v software and normalized to β-actin. Data are expressed as mean ± SD (*n* = 3). Statistical significance compared with control is indicated as *p* < 0.05 (*) and *p* < 0.01 (**).

Western blot analysis of caspase-3 revealed increased levels of cleaved caspase-3 in D-17 cells treated with cisplatin (Figure 8). Intensity quantification of cleaved caspase-3 was normalized to β-actin. Cisplatin treatment alone resulted in a dose-dependent increase in cleaved caspase-3 expression, with a statistically significant elevation observed at 1.5 mM (*p* = 0.0123; mean intensity = 0.5073 ± 0.1356 SEM). Although lower concentrations did not reach statistical significance, a biological trend toward increased cleaved caspase-3 activation was evident at concentrations of 1,000 μM and above. In contrast, combination treatment demonstrated a biological trend toward increased cleaved caspase-3 activation at lower cisplatin doses compared with untreated controls (Figure 8).

**Figure 9:**
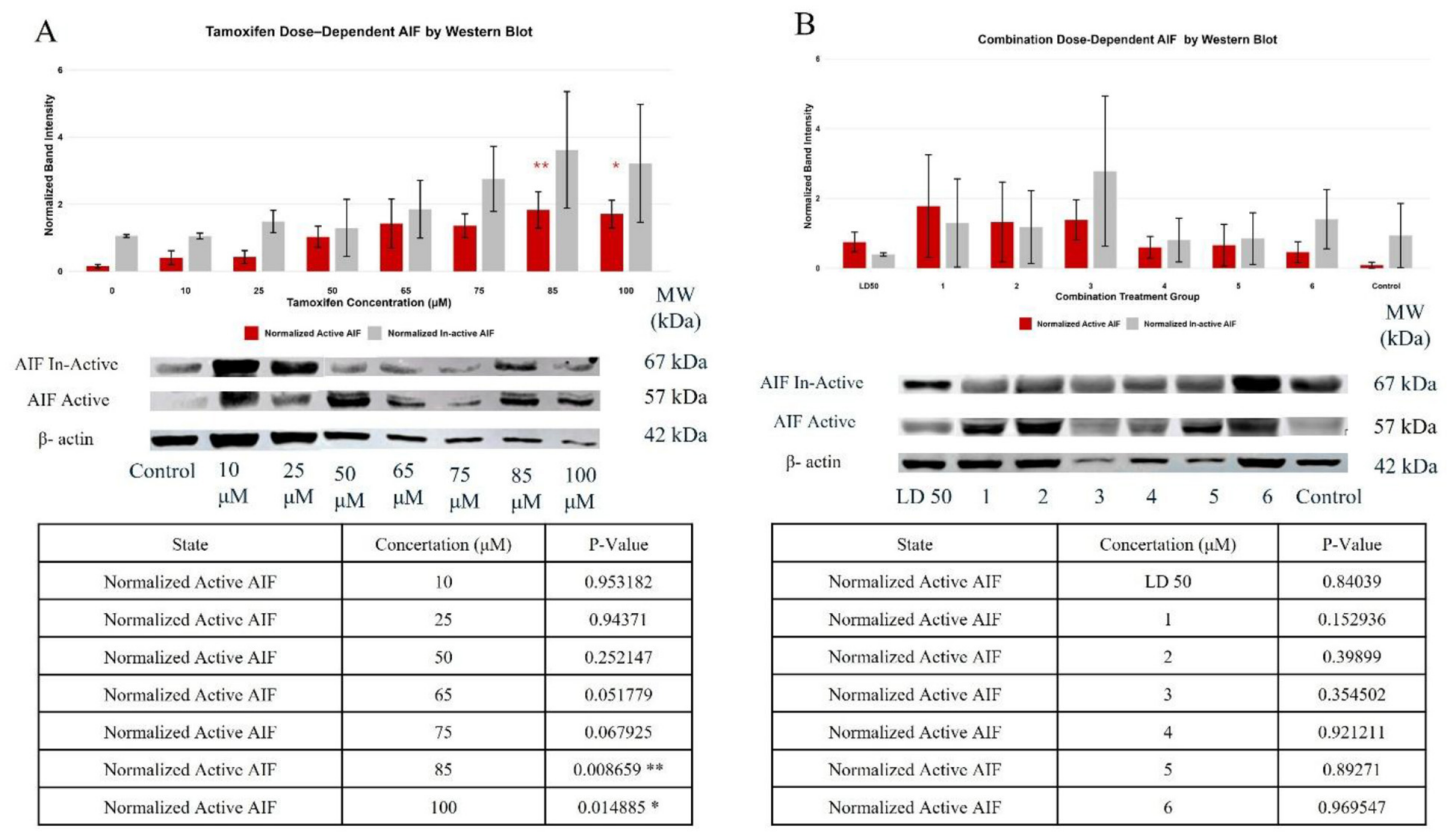
Effects of tamoxifen alone and tamoxifen in combination with cisplatin on apoptosis-inducing factor (AIF) expression in D-17 cells. Cells were treated with tamoxifen alone or with tamoxifen and cisplatin in combination for 24 h. Untreated cells served as controls. Western blot analysis was performed to assess active and inactive apoptosis-inducing factor (AIF) expression, with β-actin used as a loading control. **(A)** Representative western blots and densitometric analysis of active AIF following tamoxifen treatment alone. **(B)** Representative western blots and densitometric analysis of active AIF following combination treatment with tamoxifen and cisplatin. Band intensities were quantified using U-Scan-It Gel 7.1v software and normalized to β-actin. Data are expressed as mean ± SEM (*n* = 3). Statistical significance compared with control is indicated as *p* < 0.05 (*) and *p* < 0.01 (**).

Western blot analysis was performed to evaluate AIF expression in D-17 cells following treatment with tamoxifen alone and in combination with cisplatin (Figure 9). Intensity quantification of active AIF was normalized to β-actin. Tamoxifen treatment alone resulted in a dose-dependent increase in active AIF expression. A statistically significant increase was observed at 85 μM (*p* = 0.0087; mean intensity = 1.6770 ± 0.4272 SEM), with active AIF levels remaining significantly elevated at 100 μM (*p* = 0.0149; mean intensity = 1.5555 ± 0.4272 SEM). Although lower concentrations did not reach statistical significance, a biological trend toward increased AIF activation was evident starting at approximately 50 μM, which may indicate dose-dependent activation of AIF at higher tamoxifen concentrations. In combination treatment with cisplatin and tamoxifen a biological trend toward increased AIF activation was observed across several combination treatment groups.

**Figure 10:**
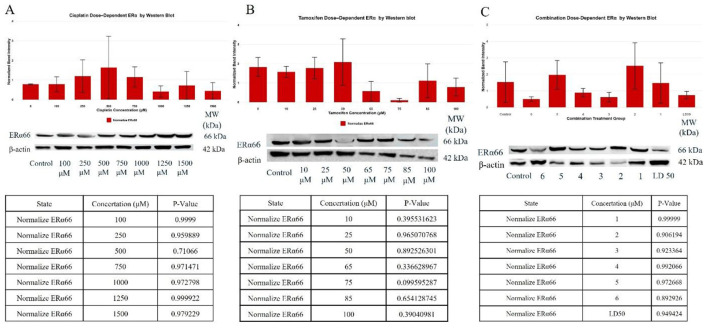
Effects of tamoxifen alone and tamoxifen in combination with cisplatin on estrogen receptor alpha (ERα66) expression in D-17 cells. Western blot analysis was performed to assess estrogen receptor alpha (ERα66) expression, with β-actin used as a loading control. **(A)** Representative western blots and densitometric analysis of ERα66 following cisplatin treatment. **(B)** Representative western blots and densitometric analysis of ERα66 following tamoxifen treatment. **(C)** Representative western blots and densitometric analysis of ERα66 following combined tamoxifen and cisplatin treatment. Band intensities were quantified using U-Scan-It Gel 7.1v software and normalized to β-actin. Data are expressed as mean ± SEM (*n* = 3). Statistical significance compared with control is indicated as *p* < 0.05 (*) and *p* < 0.01 (**).

Western blot analysis was performed to evaluate ERα expression in D-17 cells following treatment with cisplatin and tamoxifen individually and in combination (Figure 10). Intensity quantification was normalized to β-actin. Treatment with cisplatin alone did not result in statistically significant changes in ERα66 protein expression across the range of concentrations tested, and no consistent dose-dependent or biologically meaningful trends were observed compared with untreated controls. Similarly, tamoxifen treatment alone produced no significant alterations in ERα66 expression, despite minor variability between individual doses. Although isolated concentrations showed modest numerical fluctuations, these changes were not consistently seen and no trends were observed. Combination treatment with cisplatin and tamoxifen similarly did not induce significant changes in ERα66 protein expression relative to controls. No statistically significant differences or trends were detected across combination treatment groups.

**Figure 11:**
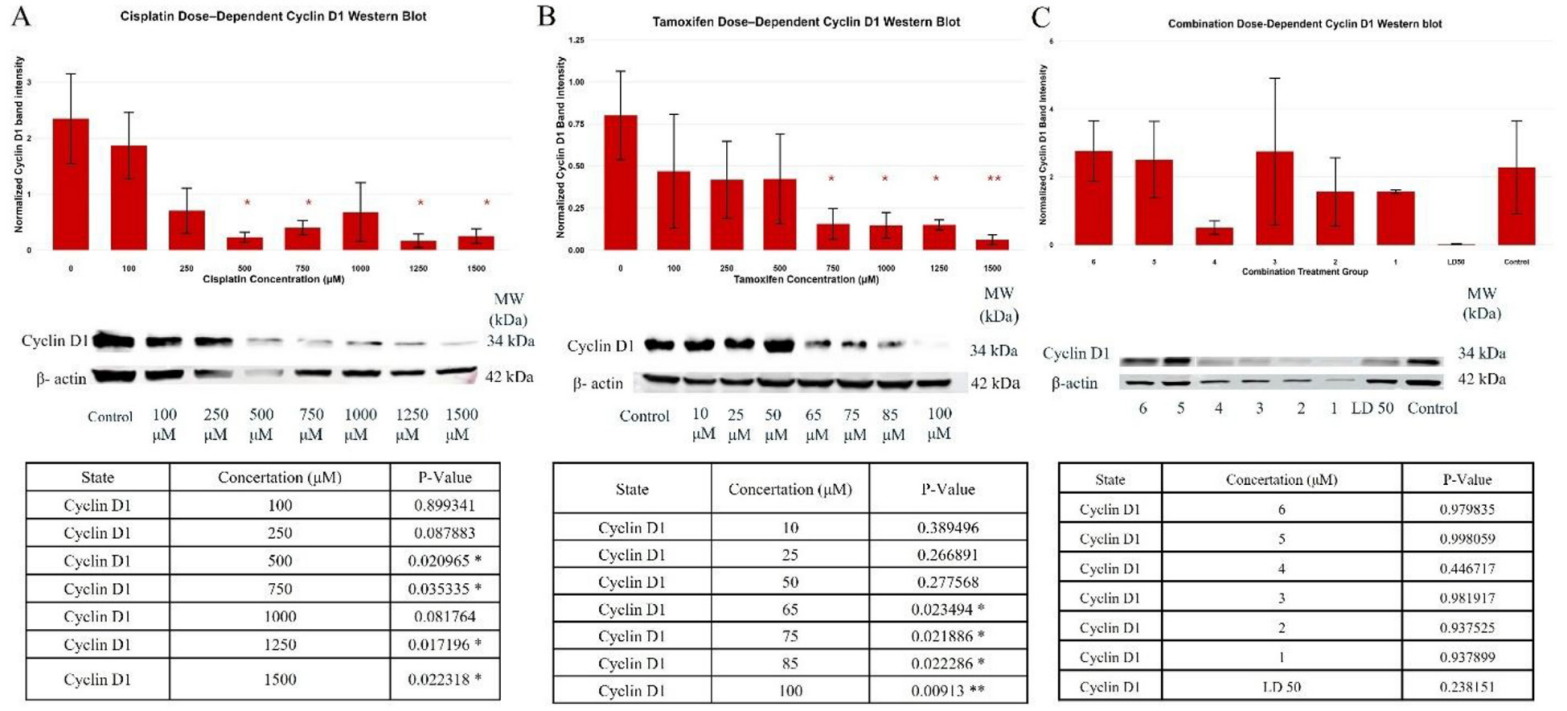
Effects of cisplatin, tamoxifen, and combination treatment on Cyclin D1 expression in D-17 cells. Western blot analysis was performed to assess Cyclin D1 expression, with β-actin used as a loading control. **(A)** Representative western blots and densitometric analysis of Cyclin D1 following cisplatin treatment. **(B)** Representative western blots and densitometric analysis of Cyclin D1 following tamoxifen treatment. **(C)** Representative western blots and densitometric analysis of Cyclin D1 following combined tamoxifen and cisplatin treatment. Band intensities were quantified using U-Scan-It Gel 7.1v software and normalized to β-actin. Data are expressed as mean ± SEM (*n* = 3). Statistical significance compared with control is indicated as *p* < 0.05 (*) and *p* < 0.01 (**).

Western blot analysis was performed to assess Cyclin D1 expression in D-17 cells following treatment with cisplatin alone, tamoxifen alone, or a combination of cisplatin and tamoxifen (Figure 11). Intensity of the band quantification of Cyclin D1 was normalized to β-actin.

Cisplatin individual treatment resulted in a dose-dependent reduction in Cyclin D1 expression. Statistically significant decreases were observed at 500 μM (*p* = 0.0209; mean intensity = −2.1140 ± 0.6107 SEM), 750 μM (*p* = 0.0353; mean intensity = −1.9451 ± 0.6107 SEM), 1,250 μM (*p* = 0.0172; mean intensity = −2.1777 ± 0.6107 SEM), and 1,500 μM (*p* = 0.0223; mean intensity = −2.0938 ± 0.6107 SEM). Although not all intermediate concentrations reached statistical significance, a consistent trend toward decreased Cyclin D1 expression was evident beginning at 500 μM.

Tamoxifen treatment alone similarly resulted in a dose-dependent decrease in Cyclin D1 expression. Statistically significant reductions were observed at 65 μM (*p* = 0.0235; mean intensity = −0.6461 ± 0.1900 SEM), 75 μM (*p* = 0.0219; mean intensity = −0.6532 ± 0.1900 SEM), 85 μM (*p* = 0.0223; mean intensity = −0.6514 ± 0.1900 SEM), and 100 μM (*p* = 0.0091; mean intensity = −0.7405 ± 0.1900 SEM). Lower concentrations did not reach statistical significance, however, a trend toward decreasing Cyclin D1 expression was apparent with increasing tamoxifen doses. Although combination treatment with cisplatin and tamoxifen did not produce statistically significant changes in Cyclin D1 expression, a biological trend toward decreased expression was evident across treatment groups.

## 4 Discussion

Tamoxifen was first discovered in the late 1960s and has since significantly improved the treatment of human breast cancer (36, 37). Approximately 70%−80% of breast cancers are estrogen receptor (ER)-positive, and tamoxifen binds these receptors to inhibit estrogen-driven proliferation (38). Studies from human and canine research suggest that sex hormones may influence OSA, although the relationship between OSA and ERα expression remains unclear due to limited research in the field (22, 39). In human OSA, some studies indicate that ERα expression is a critical risk factor, while more recent analyses found no detectable levels of ERα (22, 40–45). In canine OSA, a recent study reported that ERα was the most abundantly expressed receptor in osteoblastic tumors taken from biopsy samples, the most common subtype of OSA (46, 47). In contrast, analysis of three OSA cell lines revealed that Progesterone (PR) was the most highly expressed receptor and ERα was present at low levels (46).

Based on previous studies, the prevalence of ERα expression was assessed in Abrams D-17 canine OSA cells to evaluate both baseline expression and the effects of tamoxifen. Western blot and immunofluorescence assays were used to assess ERα expression levels. No observable changes in staining intensity were observed by immunofluorescence, and no significant differences in protein expression were detected by western blot following treatment compared with untreated controls. These findings may suggest that the observed cytotoxic effects are mediated through estrogen-independent mechanisms. However, it is important to note that the difference between ERα expression *in vivo* and vitro samples has been shown to be inconsistent. Some studies have shown that *in vitro* osteoblastic samples expressing ERα do not exhibit the same level of expression in similarly tested *in vivo* samples (22, 45, 46). This could indicate that estrogen receptor signaling in cell lines may be inconsistent and may not represent signaling within actual tumor tissue. Consequently, the *in vitro* synergy observed between tamoxifen and cisplatin may reflect the limited efficacy of tamoxifen's estrogen receptor inhibition effects that potentially could be observed *in vivo* drug therapy.

Additionally, tamoxifen has been reported to induce cell death through both estrogen receptor (ER) dependent and independent mechanisms (48). While ER-dependent signaling most commonly influences upstream apoptotic pathways, the activation of apoptosis-inducing factor (AIF) is more commonly associated with mitochondrial stress and dysfunction from downstream cascades (49). Because AIF is released from the mitochondria during downstream signaling events and mediates caspase-independent apoptosis, its involvement supports activation of mitochondrial apoptotic pathways. However, AIF activation does not definitively distinguish between ER-dependent and ER-independent mechanisms, as ER signaling can, in some contexts, influence mitochondrial function and apoptotic regulation pathways upstream. Therefore, upstream ER-dependent effects cannot be excluded. Further studies are needed to fully understand the effects of tamoxifen's estrogen dependent and independent properties on OSA and to provide evidence supporting the exact mechanism enhancing the efficacy of cisplatin.

When evaluated as individual treatment, both cisplatin and tamoxifen demonstrated time dependent cytotoxic effects in D17 OSA cells but with different potencies. Cisplatin's LD50 declined from 3,863 μM at 8 h to 68 μM at 48 h, indicating limited early toxicity, progressively increased effectiveness, and a trend consistent with resistance (Figure 2). Although there is no universally accepted numerical range to define cisplatin resistance, LD50 values in the tens to hundreds of micromolar range have been interpreted as relatively insensitive *in vitro* compared with sensitive lines that exhibit lower micromolar LD50 values in other OSA models (50–53).

In contrast, tamoxifen produced a more rapid and pronounced reduction in viability at substantially lower concentrations, with LD50 values declining from 95.11 μM to 48.16 μM over 48 h (Figure 3). The relatively lower concentration range of tamoxifen's LD_50_, and the progressive decrease over time, indicate a greater intrinsic cytotoxicity in this model compared with cisplatin. Likewise, cisplatin exhibited limited immediate cytotoxicity at early time points and required roughly 4 times higher concentrations than tamoxifen to achieve the same LD50 at 24 h, whereas tamoxifen's effects were evident at substantially lower doses and accumulated rapidly with prolonged exposure. Together, these individual evaluations provide important context for interpreting combination therapy results.

ZIP synergy scoring revealed that the combination of tamoxifen and cisplatin produced synergistic inhibition of OSA cell viability. Initially, synergy was most pronounced at higher tamoxifen concentrations combined with moderate to high cisplatin doses, as highlighted by dose-response matrices and heatmaps. Overall synergy scores increased over time, from 7.63 at 12 h to 14.39 at 48 h, with scores around 0 considered additive and values above 10 indicates synergy (54) (Figure 4). At 12 h, synergistic effects were observed only at the highest cisplatin concentration and intermediate tamoxifen dosage. By 24 and 48 h, the regions of synergy expanded broader, with lower concentrations of both drugs producing substantial reductions in metabolic activity that were not observed with individual treatments. For example, at 24 h, the combination of 151 μM cisplatin and 52 μM tamoxifen resulted in ~50% inhibition, whereas cisplatin alone required 240.40 μM to achieve 50% cell death at 24 h (Figure 4). These findings demonstrate a clear synergistic interaction between the two agents, suggesting that tamoxifen potentiates cisplatin. To better understand the synergistic mechanism of this combination, apoptotic and proliferation markers were analyzed following individual and combined treatments.

Caspase-3 exists as an inactive precursor in the cytoplasm and mitochondria of cells and becomes activated through cleavage following cellular damage (55, 56). Once activated, cleaved caspase-3 drives apoptotic disassembly of the cell which can be useful in measuring death of cells. Caspase-3 was activated in cells treated with cisplatin, as demonstrated visually by immunofluorescence assay and western blot analysis (Figures 5, 8). Western blot analysis revealed caspase-3 activation at concentrations ≥1 mM, a finding that aligns with results from the RealTime-Glo viability assay, which identified an LD50 of 859 μM (Figure 2). This data suggest that caspase-3 activation occurs near cytotoxic cisplatin concentrations. In contrast, tamoxifen-treated cells did not exhibit cleaved caspase-3 but showed elevated levels of apoptosis-inducing factor (AIF) (Figures 6, 9). Inactive AIF resides in the mitochondria and becomes activated under severe oxidative stress (57, 58). Once triggered, AIF translocated to the nucleus and induces cell death through caspase-independent pathways.

In combinational treatment cleaved caspase-3 was expressed at cisplatin concentrations lower than those required for individual cisplatin treatment. Additionally, AIF expression levels visually remained consistently elevated during combination treatment (Figures 7, 9). Although this trend on western blot did not reach statistical significance, it may suggest a potential contribution of AIF-mediated apoptosis during combination treatment. Collectively, these findings indicate that tamoxifen may help overcome cisplatin resistance and that the combination therapy engages complementary apoptotic pathways to enhance anti-tumor efficacy.

Ki-67 is a protein expressed in actively dividing cells and is present during all phases of the cell cycle except G0 phase and is commonly found at higher levels in cancer cells (59, 60). In D-17 cells Ki-67 showed an observable reduction in expression following treatment with cisplatin, tamoxifen, and particularly their combination, compared with untreated controls (Figures 5–7) (60). Similarly, Cyclin D1, a key regulator of the G1/S phase transition, showed decreased expression under the same treatment conditions (Figure 11) (61). The decreases observed with combination therapy for both Ki-67 and Cyclin D1 further suggest a synergistic effect of cisplatin and tamoxifen inhibiting OSA cell proliferation. Although no statistically significant differences were observed on western blot, a trend toward decreased Cyclin D1 expression was noted in the combination treatment groups. This trend was observed at lower doses of each agent after 24 h of treatment, that may indicate combination therapy decreases Cyclin D1 expression at reduced drug concentrations.

While our study provides valuable insights, several limitations should be acknowledged. Experiments were conducted *in vitro* using a limited number of cell lines, which may not fully capture the heterogeneity of OSA *in vivo*. Although we observed no change in estrogen expression tamoxifen could still exhibit estrogen-dependent effects, however the precise molecular pathways responsible were not fully demonstrated. Dose-response relationships and potential toxicity *in vivo* were not assessed, limiting clinical insight. Future studies should incorporate multiple canine OSA models and include *in vivo* validation of the efficacy and safety of combination therapy. Additionally, the assessment of resistance markers will be essential to further support mechanistic studies and precisely define the molecular pathways through which tamoxifen exerts its cytotoxic effects in canine OSA.

In summary, our findings indicate that tamoxifen and cisplatin induce cytotoxicity in canine osteosarcoma cells through multiple distinct apoptotic pathways. Their combined use enhances antitumor activity compared with either agent alone, supporting tamoxifen's potential role as a novel adjuvant to conventional chemotherapy. These results provide a foundation for further preclinical investigation of combination strategies in canine osteosarcoma.

## Data Availability

The raw data supporting the conclusions of this article will be made available by the authors, without undue reservation.
